# Correlation Between Fasting Blood Glucose and Serum Lipid Profile and Lipid Ratios in Patients with Type 2 Diabetes Mellitus in Southern Thailand: A Cross-Sectional Study

**DOI:** 10.3390/medsci14020302

**Published:** 2026-06-11

**Authors:** Naparat Sukkriang, Suttida Sangpoom, Sumet Khumphairan

**Affiliations:** 1 School of Medicine, Walailak University, Nakhon Si Thammarat 80160, Thailand; 2 Walailak University Hospital, Nakhon Si Thammarat 80160, Thailand; 3 Center of Excellence in Data Science for Health Science, Division of Mathematics and Statistics, School of Science, Walailak University, Nakhon Si Thammarat 80160, Thailand; suttida.sa@mail.wu.ac.th; 4 Institute of Mathematics, 69120 Heidelberg, Germany; sumet.khumphairan@gmail.com

**Keywords:** lipid ratios, blood glucose, lipid levels, type 2 diabetes mellitus, Thailand

## Abstract

**Background/Objectives:** This analysis aims to examine the correlations involving glucose levels, serum lipid parameters, and lipid ratios in adults diagnosed with type 2 diabetes mellitus (T2DM) in Southern Thailand. **Methods:** A cross-sectional investigation included 360 individuals diagnosed with T2DM. Associations among fasting blood glucose (FBG), HbA1c, lipid variables, TG/HDL, TyG, and TyG-BMI were assessed using Spearman correlation analysis. Linear and logistic regression models were applied to identify factors related to TG/HDL and elevated TG/HDL (≥3.5). Receiver operating characteristic analysis evaluated marker performance for poor glycemic control. **Results:** HbA1c showed more consistent associations with atherogenic lipid indices than FBG. TyG demonstrated the strongest association with glycemic status and the highest discriminatory performance for HbA1c ≥ 7%. In adjusted analyses, HbA1c and BMI remained independently associated with higher TG/HDL and increased odds of TG/HDL. **Conclusions:** HbA1c was more strongly associated with atherogenic lipid abnormalities than FBG. Among the evaluated lipid-related indices, TG/HDL and TyG showed the closest relationships with glycemic status, while TyG demonstrated the highest ability to discriminate poor glycemic control.

## 1. Introduction

Type 2 Diabetes Mellitus (T2DM) is a worldwide chronic disease that markedly affects the quality of life [1]. It results from diminished pancreatic β-cell insulin secretion and peripheral insulin resistance, leading to chronic hyperglycemia [2]. Persistent hyperglycemia is a key contributor to cardiovascular disease (CVD), promoting inflammation, oxidative stress, and structural vascular remodeling, and inducing dyslipidemia characterized by elevated triglycerides (TG) and reduced high-density lipoprotein cholesterol (HDL), thereby accelerating atherosclerosis and significantly increasing cardiovascular morbidity and mortality [3].

Chronic hyperglycemia contributes to pancreatic β-cell dysfunction and oxidative stress, promoting the formation of oxidized low-density lipoprotein (LDL) and accelerating atherogenesis. Mixed dyslipidemia—such as high TG, low HDL, and small, dense LDL particles—affects up to 80–90% of patients with T2DM [4]. The development of atherosclerotic CVD is greatly influenced by this lipid pattern, which is strongly associated with insulin resistance [5]. Notably, the presence of diabetic complications such as retinopathy and nephropathy, despite conventional glycemic control, highlights the importance of factors beyond blood glucose in determining microvascular risk [6]. Complex, multifactorial mechanisms—including chronic hypertension, dyslipidemia, inflammation, oxidative stress, and structural cellular injury—drive these complications.

Based on recommendations from the American Diabetes Association (ADA) guidelines, glycemic control in patients with T2DM is commonly assessed using FBG and HbA1c. Poor glycemic control is generally defined as FBG > 126 mg/dL or HbA1c > 7% [7]. Nonetheless, the reliability of HbA1c can be influenced by a variety of diseases, such as end-stage kidney disease (ESRD), iron deficiency anemia, vitamin B12 deficiency, thalassemia, acute blood loss, hemolytic anemia, recent blood transfusion, liver cirrhosis, and medications such as vitamin C, vitamin E, dapsone, ribavirin, and hydroxyurea [8]. These limitations highlight the importance of complementary metabolic markers that may better reflect ongoing metabolic dysregulation.

Lipid parameters, including total cholesterol (TC), serum TG, and LDL, are commonly applied to assess CVD risk in clinical practice. In addition, lipid ratios such as TC/HDL, TG/HDL, and LDL/HDL are increasingly recognized as useful indicators integrating multiple lipid measures for improved assessment of CVD risk [9]. Non-HDL, which indicates the total burden of atherogenic lipoproteins, is increasingly recognized as a valuable indicator of CVD risk. Furthermore, the atherogenic index of plasma (AIP) derived from the base-10 logarithm of the TG/HDL has emerged as another marker associated with CVD risk.

Recent evidence indicates that glucose dysregulation and lipid abnormalities are closely interconnected and are both associated with increased cardiometabolic risk [10]. Previous studies have examined these associations in Asian populations; however, direct comparisons of FBG, HbA1c, conventional lipid parameters, lipid ratios, and TyG-related indices within the same clinical cohort remain scarce [11,12,13,14]. Therefore, this study aimed to evaluate these relationships in Southern Thai patients with T2DM.

This analysis aims to examine the correlations involving FBG and serum lipid levels and lipid ratios in adults diagnosed with T2DM in southern Thailand. By clarifying the relationship between glycemic status and atherogenic lipid profiles, this study could provide real-world evidence supporting the potential role of lipid ratios as accessible metabolic markers in routine clinical practice and inform strategies to decrease the burden of long-term complications and decrease mortality among patients with T2DM.

## 2. Research Methods

### 2.1. Research Design

This cross-sectional investigation was performed between 1 July 2025 and 1 March 2026 at the Internal Medicine Outpatient Clinic, Walailak University Hospital, Thasala District, Nakhon Si Thammarat, southern Thailand. Data were collected from patients attending routine follow-up visits for T2DM. Eligible patients were invited to participate through clinic-distributed informational leaflets describing the study objectives and procedures. The study adhered to the Declaration of Helsinki and was approved by the Research Ethics Committee on Human Rights Related to Research Involving Human Subjects, Walailak University, Thailand (approval number: WUEC-25-077-01; approved on 6 March 2025). Written informed consent was obtained from all participants before study participation.

### 2.2. Participant Enrollment and Data Collection

Adults aged ≥35 years with a confirmed diagnosis of T2DM based on ADA criteria were included in the study. Participants were excluded if they were pregnant or lacked HbA1c results within the previous 6 months. To minimize bias in glycemic assessment, patients with conditions known to interfere with HbA1c interpretation were also excluded. Exclusion criteria included ESRD (estimated glomerular filtration rate, eGFR < 15 mL/min/1.73 m^2^) or current hemodialysis, iron deficiency, vitamin B12 deficiency, thalassemia major, acute bleeding, hemolysis, recent blood transfusion (within 6 months), and liver cirrhosis, identified using ICD-10 codes. In addition, patients who had received medications known to affect HbA1c levels—such as vitamin C, vitamin E, dapsone, ribavirin, hydroxyurea, or trimethoprim/sulfamethoxazole—within the past 6 months were excluded. All participants were assessed for eligibility before enrollment.

Data were collected using a structured case record form. Demographic characteristics, clinical history, comorbidities, diabetic complications, medication use, and laboratory findings were obtained from electronic medical records and direct patient interviews. Baseline demographic and clinical variables included age, sex, BMI, heart rate, blood pressure, smoking behavior, alcohol use, and duration of T2DM. Assessed comorbidities included hypertension, dyslipidemia, coronary artery disease, and cerebrovascular disease. Diabetic complications included diabetic retinopathy, albuminuria, diabetic neuropathy, diabetic foot ulcer, diabetic ketoacidosis, hyperosmolar hyperglycemic state (HHS), and a history of hypoglycemia. Biochemical parameters were obtained from routine clinical laboratory evaluations and included FBG, HbA1c, TC, TG, HDL, LDL, kidney function indicators, and spot urine albumin-to-creatinine ratio (UACR). All measurements were performed on fasting blood samples collected during routine follow-up visits.

### 2.3. Definition of Lipid Ratios

Lipid ratios were generated to assess atherogenic lipid profiles. TC/HDL was derived from TC relative to HDL [15]. The LDL/HDL was derived from LDL relative to HDL. Non-HDL was obtained by excluding HDL from TC. The AIP was defined as the base-10 log of TG/HDL [16]. The triglyceride-glucose index (TyG) was calculated as ln [TG (mg/dL) × FPG (mg/dL)/2]. The TyG-BMI index was obtained by multiplying the TyG index by BMI. The TyG-BMI combines adiposity and glucose–lipid metabolism and has been investigated as a proxy indicator of insulin resistance [10]. In addition, the non-HDL/HDL was calculated as non-HDL divided by HDL. It has been utilized as an indirect measure of insulin resistance and metabolic dysfunction in epidemiological studies. A TG/HDL ≥ 3.5 was selected based on previous evidence linking elevated TG/HDL to increased cardiometabolic risk [17]. Although ethnicity-specific cutoffs have not been fully established in Southeast Asian populations, findings from a Taiwanese cohort support the association between higher TG/HDL and insulin resistance in an Asian population [18]. All lipid parameters were measured in mg/dL, and ratios were calculated using fasting laboratory values obtained during routine clinical assessments.

### 2.4. Statistical Analysis

The population proportion formula was applied to estimate the required sample size [19]. The prevalence (P) of dyslipidemia in Thailand was assumed to be 66.5% based on previous epidemiological data [20]. Calculations were performed using a confidence level corresponding to Z = 1.96 with a precision level of 0.05. The minimum required sample size was 343 individuals. To account for potential missing or incomplete data, the sample size was increased by 5%. The final estimated sample size was adjusted to 360 individuals. Data analyses were carried out using IBM SPSS Statistics version 29 (IBM Corp., Armonk, NY, USA). Continuous measures were presented as mean ± standard deviation or median with interquartile range, depending on data distribution, whereas categorical data were reported as frequencies and percentages. Group comparisons (Table 1 and Table 2) were evaluated using an independent *t*-test or Mann–Whitney U tests for continuous data and the chi-square or Fisher’s exact analyses for categorical data. Correlations between glycemic parameters (FBG and HbA1c) and lipid profiles (Table 3) were investigated through Spearman’s rank correlation. Variables linked to the TG/HDL were explored using univariable and multivariable linear regression analyses (Table 4 and Table 5). Covariates showing *p* < 0.20 during univariable analysis were selected for the multivariable model. Medication variables with very low prevalence were not entered into regression analyses. Specifically, bempedoic acid was excluded because fewer than 10 participants were receiving this treatment. Multicollinearity was assessed using variance inflation factors. The TG/HDL was further categorized (≥3.5) [17,21] to define high-risk status, and logistic regression analysis (Table 6) was conducted to identify related factors, with results presented as odds ratios and 95% confidence intervals. A significance level of *p* < 0.05 was applied. Linear regression assumptions were assessed using standardized residual histograms, normal P–P plots, and residual-versus-predicted value scatterplots. The assumptions of normality and homoscedasticity were considered acceptable.

## 3. Results

### Participant Characteristics

Participant screening was initially conducted in 365 individuals. All participants met the inclusion criteria of T2DM and were aged ≥35 years. After applying the exclusion criteria based on medical history, medication review, and electronic medical records, five patients were excluded due to iron deficiency (ICD-10), including two males and three females. The final study population included 360 participants, comprising 188 females (52.2%) and 172 males (47.8%). Among them, two females and two males were newly diagnosed with T2DM, while the remaining participants had established disease and were undergoing routine follow-up care at the outpatient medical clinic.

Table 1 presents the baseline characteristics of the enrolled individuals. No sex significant differences were observed in age, BMI, blood pressure, heart rate, or duration of diabetes. Males had significantly higher body weight and height than females (both *p* < 0.001). In contrast, lifestyle factors differed significantly between sexes, with smoking and alcohol consumption being significantly more prevalent among males (both *p* < 0.001). Diabetes duration categories were similar across both sexes (*p* = 0.395). The majority of patients had long-standing T2DM, with over one-third having a disease duration > 10 years (39.4% in females and 33.7% in males), while approximately 40–44% had a duration of 1–5 years. Only a small proportion had a duration < 1 year. Hypertension prevalence was greater in females than males (75.5% vs. 65.1%, *p* = 0.030), whereas dyslipidemia, coronary artery disease, and stroke were similar between groups. Similarly, diabetes-related complications—retinopathy, neuropathy, foot ulcers, diabetic ketoacidosis, and recent hypoglycemia—showed comparable distributions between females and males. Neither male nor female participants had HHS. The prevalence of albuminuria (≥30 mg/g) was similar in females and males (37.8% vs. 39.5%, *p* = 0.731). Among newly diagnosed patients (two males and two females), screening for diabetic retinopathy and foot examination revealed no abnormalities. Medication use did not differ significantly between sexes across all classes of antidiabetic and lipid-lowering agents.

Table 2 summarizes the laboratory characteristics of the study participants. FBG and HbA1c values were comparable between females and males. Hemoglobin levels were higher in males than in females, whereas TC and HDL levels were higher among female participants (both *p* ≤ 0.001). Non-HDL was also slightly higher in females (*p* = 0.031), while LDL showed a borderline but non-significant difference (*p* = 0.054). TG concentrations did not differ between groups. Similarly, lipid ratios, including TC/HDL, TG/HDL, LDL/HDL, non-HDL/HDL, TyG, TyG-BMI, and AIP, were comparable between sexes. Regarding renal parameters, eGFR was higher in females (*p* = 0.001), whereas UACR did not differ significantly between groups.

Table 3 summarizes the relationships between glycemic markers and lipid-related parameters. Positive associations were observed between both FBG and HbA1c and most lipid measures. Among the lipid ratios, non-HDL/HDL exhibited the highest correlation with HbA1c (*r* = 0.20, *p* < 0.001). TyG showed the strongest associations with both FBG (*r* = 0.62, *p* < 0.001) and HbA1c (*r* = 0.42, *p* < 0.001). Correlation coefficients were generally higher for HbA1c than for FBG across several lipid ratios. In contrast, TyG-BMI displayed only a weak association with FBG (*r* = 0.18, *p* = 0.001) and was not significantly related to HbA1c (*p* = 0.157). HDL was inversely associated with HbA1c, whereas no significant relationship was observed with FBG.

Table 4 summarizes the discriminatory performance of lipid parameters and lipid-related indices for identifying poor glycemic control (HbA1c ≥ 7%). Among all evaluated markers, TyG demonstrated the highest area under the curve. Conventional lipid parameters and lipid ratios showed lower discriminatory ability, with AUC values ranging from 0.448 to 0.576. TyG-BMI was not significantly associated with poor glycemic control. Overall, TyG exhibited greater discriminatory performance than conventional lipid parameters and lipid ratios for identifying poor glycemic control in this study.

Table 5 presents the univariable and multivariable linear regression analyses for TG/HDL. In the adjusted model, HbA1c, BMI, male sex, insulin therapy, fibrate use, and eicosapentaenoic acid use remained significantly associated with higher TG/HDL values. In contrast, age, duration of diabetes, metformin use, and statin therapy were not independently associated with TG/HDL after adjustment.

Table 6 summarizes the multivariable logistic regression analysis for TG/HDL ≥ 3.5. HbA1c and BMI remained significantly associated with elevated TG/HDL after adjustment. Insulin use, fibrate therapy, and eicosapentaenoic acid use were also associated with TG/HDL status, whereas age did not retain statistical significance in the final model.

## 4. Discussion

Our findings demonstrated significant associations between glycemic parameters and several conventional lipid measures, lipid ratios, and triglyceride-based indices. Among the evaluated markers, TyG showed the strongest correlations with both FBG and HbA1c, whereas TG/HDL, LDL/HDL, non-HDL/HDL, and AIP exhibited weaker but significant associations. Overall, HbA1c demonstrated more consistent relationships with lipid-related markers than FBG, supporting the close link between chronic glycemic exposure and atherogenic dyslipidemia. HbA1c may demonstrate stronger associations with lipid abnormalities than FBG because it reflects cumulative glycemic exposure over the preceding several months. Persistent hyperglycemia is closely linked to insulin resistance and the characteristic pattern of diabetic dyslipidemia, including elevated TG and reduced HDL, which collectively contribute to a more atherogenic lipid profile [4,5]. These findings are consistent with studies among Pakistani and Taiwanese populations. For example, research from Pakistan reported that HbA1c was closely related to TG levels and TG/HDL, with correlation coefficients of 0.977 and 0.813, respectively [22]. A study conducted in Taiwan identified that the TG/HDL is a surrogate marker of insulin resistance and suggested a threshold of approximately 3.0–3.5 for detecting elevated cardiometabolic risk [18]. A prospective cohort study in China reports that poor glycemic control and adverse lipid profiles were associated with an increased risk of cardiovascular events in patients with T2DM [23]. These findings may be explained by the pathophysiological roles of insulin resistance and chronic hyperglycemia in T2DM. Elevated HbA1c reflects prolonged glycemic exposure and promotes increased flux of free fatty acids from adipose tissue to the liver. This process enhances hepatic synthesis and secretion of very low-density lipoprotein, increasing circulating TG levels. Simultaneously, insulin resistance reduces lipoprotein lipase activity, impairing TG clearance and promoting hypertriglyceridemia [4]. Moreover, elevated TG-rich lipoproteins promote cholesteryl ester transfer protein-mediated TG transfer into HDL, producing TG-enriched HDL that is rapidly catabolized. This reduces HDL levels, as observed in a previous study [9]. These combined mechanisms explain the positive correlation between HbA1c and TG and the inverse correlation with HDL, contributing to a more atherogenic lipid profile [24]. Direct comparisons between AIP and other lipid ratios remain limited. However, a study in China reports that high AIP levels are associated with obstructive sleep apnea severity [25] and that AIP is a novel lipid marker for predicting CVD [26].

TyG and TyG-BMI were additionally evaluated as triglyceride-based markers of metabolic dysfunction [10]. TyG showed the strongest discrimination for poor glycemic control (HbA1c ≥ 7%). Among all evaluated lipid-related indices, TyG demonstrated the highest discriminatory ability for poor glycemic control AUC of 0.679. A comparable AUC of 0.65 has been reported in Thai patients with ischemic stroke for predicting major adverse cardiovascular events, suggesting that TyG may serve as a practical marker of metabolic risk across different clinical settings [27]. In contrast, TyG-BMI did not demonstrate superior performance compared with TyG alone in the present study. These findings are consistent with previous reports supporting the utility of TyG as a simple marker of metabolic dysfunction in T2DM.

HbA1c is significantly associated with lipid ratios such as TG/HDL. A study in Bosnia and Herzegovina reports significantly higher TG and TG/HDL in uncontrolled diabetes with HbA1c > 7% (TG: *p* < 0.001; TG/HDL: *p* = 0.003) [28]. These findings align with our results, in which HbA1c was positively correlated with the TG/HDL. A study in India shows a significant difference in the TG/HDL between prediabetes and normoglycemia (*p* < 0.001), whereas no significant correlation was observed between carotid intima-media thickness and the TG/HDL [15]. However, the lack of a significant correlation between the TG/HDL and carotid intima-media thickness in this study may reflect population characteristics and disease stage. The study included individuals with prediabetes and normoglycemia, in whom metabolic disturbances are generally less severe than in patients with established T2DM. As a result, lipid abnormalities reflected by the TG/HDL may not be sufficient to induce detectable structural vascular changes, such as increased carotid intima-media thickness. For other lipid ratios, a Chinese study reports that the LDL/HDL was significantly correlated with HbA1c (*p* = 0.003) [29], consistent with our findings. This suggests that the LDL/HDL may be a useful marker for assessing cardiovascular risk associated with dyslipidemia in patients with T2DM [29]. Another study on Chinese patients reports that blood glucose levels are positively associated with TG, LDL, and elevated TG/HDL and LDL/HDL, supporting a close relationship between glycemic control and atherogenic lipid profiles [13].

Logistic regression analysis revealed that HbA1c and BMI were independently associated with a high TG/HDL (≥3.5), while age was not significantly associated. Higher HbA1c was significantly associated with greater odds of having high TG/HDL, indicating that poorer glycemic control is associated with a more atherogenic lipid profile. This finding is consistent with that of previous studies reporting that the TG/HDL is associated with glycemic status and insulin resistance and is independently associated with HbA1c [28]. BMI was also independently associated with high TG/HDL, consistent with previous evidence indicating that adiposity is a key risk factor for elevated TG/HDL and metabolic risk. A cross-sectional study of patients with T2DM in China reports that higher BMI is significantly associated with increased TG and reduced HDL, contributing to an elevated TG/HDL and a more atherogenic lipid profile [13]. In contrast, age was not significantly associated with a high TG/HDL, suggesting that metabolic factors such as glycemic control and adiposity may have a stronger influence on lipid abnormalities than chronological age. TG/HDL may also serve as a simple surrogate marker of insulin resistance, altered lipid metabolism, and cardiometabolic risk in patients with T2DM [30].

Medication-related factors were included in the multivariable analysis. Insulin, fibrate, and eicosapentaenoic acid use were associated with high TG/HDL, whereas metformin and statin use were not. These findings may reflect underlying lipid abnormalities and treatment indications rather than direct drug effects. This study has several strengths. It included a relatively large sample size of patients with T2DM in a real-world clinical setting. Additionally, correlation, AUC, linear regression, and logistic regression analyses were performed, providing complementary information regarding the relationships between glycemic status, lipid parameters, lipid-derived indices, and high-risk TG/HDL status. Nonetheless, several limitations remain. First, causal relationships could not be established due to the cross-sectional design. Second, potential residual confounding may still exist, as dietary habits, duration of exercise, and socioeconomic status were not fully examined. Another limitation is that the study was conducted in a single tertiary care center in southern Thailand.

## 5. Conclusions

HbA1c was more strongly associated with atherogenic lipid abnormalities than FBG. Among the evaluated lipid-related indices, TG/HDL and TyG showed the closest relationships with glycemic status, while TyG demonstrated the highest ability to discriminate poor glycemic control. These findings suggest that TG/HDL and TyG may be useful markers for cardiometabolic risk assessment in T2DM.

## Figures and Tables

**Table 1:** Baseline characteristics.

Characteristics	Female (n = 188)	Male (n = 172)	*p*-Value
Age (years)	60.9 ± 12.2	60.0 ± 11.8	0.445
Weight (kg)	66.7 ± 14.6	75.8 ± 18.1	<0.001
Height (m)	1.55 ± 0.06	1.68 ± 0.07	<0.001
BMI (kg/m^2^)	26.7 (23.3–30.2)	26.1 (23.3–28.8)	0.065
SBP (mmHg)	131.7 ± 12.7	130.7 ± 12.9	0.482
DBP (mmHg)	73.2 ± 9.4	74.5 ± 9.1	0.178
Heart rate (bpm)	84.6 ± 12.8	83.2 ± 14.1	0.349
Smoking, n (%)	0 (0.0)	36 (20.9)	<0.001
Alcohol drinking, n (%)	3 (1.6)	44 (25.6)	<0.001
**Duration of T2DM, (years)**	7.0 (0.1–12.0)	6.0 (0.3–12.0)	0.408
**Duration of T2DM, categorical, n (%)**			0.395
<1 year	7 (3.7)	3 (1.7)	
1–5 years	75 (39.9)	76 (44.2)	
6–10 years	32 (17.0)	35 (20.3)	
>10 years	74 (39.4)	58 (33.7)	
**Comorbid conditions, n (%)**			
Hypertension	142 (75.5)	112 (65.1)	0.030
Dyslipidemia	179 (95.2)	162 (94.2)	0.663
Coronary artery disease	16 (8.5)	18 (10.5)	0.526
Stroke	10 (5.3)	7 (4.1)	0.577
**Diabetes complications, n (%)**			
Retinopathy	29 (15.4)	27 (15.7)	0.943
Albuminuria (≥30 mg/g)	71 (37.8)	68 (39.5)	0.731
Neuropathy	22 (11.7)	13 (7.6)	0.185
Foot ulcer	2 (1.1)	4 (2.3)	0.431
Diabetic ketoacidosis	0 (0.0)	1 (0.6)	0.478
Hypoglycemia (past 3 months)	3 (1.6)	1 (0.6)	0.624
**Current Medications, n (%)**			
Metformin	158 (84.0)	144 (83.7)	0.934
Sulfonylurea	71 (37.8)	66 (38.4)	0.906
Pioglitazone	25 (13.3)	28 (16.3)	0.425
DPP-4 inhibitors	57 (30.3)	55 (32.0)	0.734
SGLT2 inhibitors	77 (41.0)	78 (45.3)	0.401
GLP-1 Receptor agonist	54 (28.7)	48 (27.9)	0.864
Insulin	50 (26.6)	44 (25.6)	0.827
Statin	173 (92.0)	155 (90.1)	0.526
Fibrate	13 (6.9)	13 (7.6)	0.814
Eicosapentaenoic acid	17 (9.0)	15 (8.7)	0.915
Bempedoic acid	1 (0.5)	3 (1.7)	0.352
Ezetimibe	25 (13.3)	21 (12.2)	0.757

Values are reported as mean ± SD or median (Q1–Q3 interquartile range).

**Table 2:** Laboratory characteristics of study participants.

Characteristics	Female (n = 188)	Male (n = 172)	*p*-Value
FBG (mg/dL)	122 (99–144)	120 (102–145)	0.497
HbA1c (%)	7.2 (6.6–8.5)	7.3 (6.5–8.7)	0.223
Hemoglobin (g/dL)	12.9 ± 1.1	14.3 ± 2.2	<0.001
TC (mg/dL)	164 (140–187)	151 (130–177)	0.001
TG (mg/dL)	117.5 (88–151)	120 (85–165)	0.746
HDL (mg/dL)	48.7 ± 11.8	43.9 ± 11.7	<0.001
LDL (mg/dL)	92.5 (72–113)	85 (65–106)	0.054
eGFR (mL/min/1.73 m^2^)	89.4 ± 22.1	81.5 ± 23.1	0.001
UACR (mg/g)	16.7 (6.5–57.2)	18.5 (6.0–88.5)	0.752
TC/HDL	3.43 (2.85–4.58)	3.60 (2.90–4.99)	0.256
TG/HDL	2.47 (1.56–3.37)	2.67 (1.46–3.88)	0.189
LDL/HDL	1.93 (1.36–2.50)	2.07 (1.54–2.60)	0.406
Non-HDL (mg/dL)	114 (93–133)	108 (85–134)	0.031
Non-HDL/HDL	2.43 (1.85–3.01)	2.58 (1.89–3.28)	0.081
TyG	8.92 ± 0.53	8.93 ± 0.63	0.918
TyG-BMI	236.73 (212.87–274.82)	229.71 (205.53–261.58)	0.069
AIP	0.39 (0.24–0.54)	0.43 (0.24–0.62)	0.189

Values are reported as mean ± SD or median (Q1–Q3 interquartile range). TyG-BMI was calculated as TyG × BMI. AIP was expressed as log10(TG/HDL). TyG index (TyG) was calculated as ln [TG (mg/dL) × FPG (mg/dL)/2].

**Table 3:** Correlation between glycemic parameters and lipid profile (n = 360).

Variables	FBG (*r*)	*p*-Value	HbA1c (*r*)	*p*-Value
TC	0.15	0.004	0.15	0.005
TG	0.22	<0.001	0.14	0.007
LDL	0.14	0.009	0.16	0.002
HDL	−0.04	0.443	−0.13	0.013
Non-HDL	0.17	0.001	0.19	<0.001
TC/HDL	0.13	0.013	0.20	<0.001
TG/HDL	0.17	0.001	0.17	0.002
LDL/HDL	0.12	0.022	0.18	0.001
Non-HDL/HDL	0.13	0.013	0.20	<0.001
TyG	0.62	<0.001	0.42	<0.001
TyG-BMI	0.18	0.001	0.08	0.157
AIP	0.17	0.001	0.17	0.002

Spearman’s rank correlation analysis was applied to determine correlation coefficients (*r*).

**Table 4:** ROC analysis for identifying poor glycemic control (HbA1c ≥ 7%) (n = 360).

Variables	AUC (95% CI)	*p*-Value
TC	0.561 (0.501–0.621)	0.049
TG	0.550 (0.490–0.610)	0.106
HDL	0.448 (0.388–0.509)	0.097
LDL	0.565 (0.504–0.625)	0.038
Non-HDL	0.576 (0.516–0.637)	0.014
TC/HDL	0.567 (0.507–0.627)	0.031
TG/HDL	0.558 (0.499–0.618)	0.061
LDL/HDL	0.561 (0.501–0.621)	0.051
Non-HDL/HDL	0.567 (0.507–0.627)	0.031
TyG	0.679 (0.624–0.734)	<0.001
TyG-BMI	0.526 (0.466–0.586)	0.405
AIP	0.558 (0.499–0.618)	0.061

**Table 5:** Associations between clinical variables and TG/HDL (n = 360).

Variables	Univariable β (95% CI)	*p*-Value	Multivariable β (95% CI)	*p*-Value	
HbA1c (%)	0.245 (0.118–0.371)	<0.001	0.202 (0.073–0.332)	0.002	
Age (years)	−0.031 (−0.050 to −0.012)	0.002	−0.007 (−0.028–0.014)	0.502	
BMI (kg/m^2^)	0.069 (0.027–0.111)	0.001	0.056 (0.014–0.097)	0.009	
Sex (male)	0.590 (0.133–1.047)	0.012	0.567 (0.154–0.981)	0.007	
Smoking	0.325 (−0.441–1.092)	0.405	—	—	
Alcohol consumption	0.389 (−0.293–1.071)	0.263	—	—	
Duration of T2DM (years)	−0.023 (−0.052–0.006)	0.121	−0.030 (−0.062–0.001)	0.058	
Metformin	0.524 (−0.100–1.148)	0.099	0.168 (−0.420–0.755)	0.575	
Sulfonylurea	0.097 (−0.377–0.571)	0.687	—	—	
Pioglitazone	0.351 (−0.297–1.000)	0.288	—	—	
DPP-4 inhibitor	−0.239 (−0.735–0.258)	0.346	—	—	
SGLT2 inhibitor	−0.098 (−0.563–0.367)	0.678	—	—	
GLP-1 receptor agonist	0.117 (−0.394–0.628)	0.652	—	—	
Insulin	0.794 (0.276–1.312)	0.003	0.730 (0.173–1.287)	0.010	0.009
Statin	−0.545 (−1.352–0.262)	0.185	−0.207 (−0.935–0.520)	0.576	0.007
Fibrate	2.739 (1.896–3.582)	<0.001	1.928 (1.078–2.779)	<0.001	0.058
Ezetimibe	0.159 (−0.531–0.848)	0.651	—	—	0.575
Eicosapentaenoic acid	1.899 (1.114–2.683)	<0.001	1.371 (0.603–2.138)	0.001	0.010

Variables with *p* < 0.20 were entered into the multivariable model. β, unstandardized coefficient.

**Table 6:** Multivariable logistic regression analysis (≥3.5) (n = 360).

Variables	OR	95% CI	*p*-Value
HbA1c (%)	1.19	1.03–1.38	0.022
Age (years)	0.99	0.96–1.01	0.315
BMI (kg/m^2^)	1.06	1.01–1.12	0.015
Sex (male)	1.40	0.85–2.29	0.186
Duration of T2DM (years)	0.98	0.94–1.02	0.311
Metformin	1.03	0.50–2.11	0.942
Insulin	2.12	1.11–4.07	0.023
Statin	0.68	0.30–1.55	0.356
Fibrate	2.89	1.08–7.71	0.034
Eicosapentaenoic acid	5.50	2.29–13.18	<0.001

ORs were calculated from multivariable logistic regression.

## Data Availability

The original contributions presented in this study are included in the article. Further inquiries can be directed to the corresponding author.

## Abbreviations

T2DM	Type 2 diabetes mellitus
FBG	Fasting blood glucose
HbA1c	Hemoglobin A1c
TC	Total cholesterol
TG	Triglycerides
HDL	High-density lipoprotein cholesterol
LDL	Low-density lipoprotein cholesterol
Non-HDL	Non–high-density lipoprotein cholesterol
TC/HDL	Total cholesterol to HDL-C ratio
TG/HDL	Triglyceride to HDL-C ratio
LDL/HDL	LDL-C to HDL-C ratio
AIP	Atherogenic index of plasma
TyG	Triglyceride-glucose index
TyG-BMI	Triglyceride-glucose body mass index
BMI	Body mass index
SBP	Systolic blood pressure
DBP	Diastolic blood pressure
eGFR	Estimated glomerular filtration rate
CI	Confidence interval
OR	Odds ratio
UACR	Urine albumin-to-creatinine ratio
AUC	Area under the curve

## References

[1] Khunkaew S., Fernandez R., Sim J. (2019). Demographic and clinical predictors of health-related quality of life among people with type 2 diabetes mellitus living in northern Thailand: A cross-sectional study. Health Qual. Life Outcomes.

[2] Młynarska E., Czarnik W., Dzieża N., Jędraszak W., Majchrowicz G., Prusinowski F., Stabrawa M., Rysz J., Franczyk B. (2025). Type 2 Diabetes Mellitus: New Pathogenetic Mechanisms, Treatment and the Most Important Complications. Int. J. Mol. Sci..

[3] Chakraborty S., Verma A., Garg R., Singh J., Verma H. (2023). Cardiometabolic Risk Factors Associated with Type 2 Diabetes Mellitus: A Mechanistic Insight. Clin. Med. Insights Endocrinol. Diabetes.

[4] Chandana M.S., Vijaykumar S., Anudhar G.P. (2025). Type 2 diabetes mellitus with dyslipidemia: Risk factors, prevalence, pathophysiology, and nutritional management-a narrative review. Int. J. Community Med. Public Health.

[5] González-Lleó A.M., Brito-Casillas Y., Martín-Santana V., Gil-Quintana Y., Tugores A., Scicali R., Riaño M., Hernández-Baraza L., Jiménez-Monzón R., Girona J. (2025). Glucose metabolism in heterozygous familial hypercholesterolemia with a founder effect and a high diabetes prevalence: A cross-sectional study. Cardiovasc. Diabetol..

[6] Vithian K., Hurel S. (2010). Microvascular complications: Pathophysiology and management. Clin. Med..

[7] American Diabetes Association Professional Practice Committee (2026). Diagnosis and Classification of Diabetes: Standards of Care in Diabetes-2026. Diabetes Care.

[8] Radin M.S. (2014). Pitfalls in hemoglobin A1c measurement: When results may be misleading. J. Gen. Intern. Med..

[9] Millán J., Pintó X., Muñoz A., Zúñiga M., Rubiés-Prat J., Pallardo L.F., Masana L., Mangas A., Hernández-Mijares A., González-Santos P. (2009). Lipoprotein ratios: Physiological significance and clinical usefulness in cardiovascular prevention. Vasc. Health Risk Manag..

[10] Du K., Zhang Y., Zhang X., Pan Y., Pan T., Tong Y., Chen X., Lv D. (2026). Associations of cholesterol, high-density lipoprotein and glucose index and triglyceride-glucose-related indices with carotid atherosclerosis in young and middle-aged individuals. Front. Endocrinol..

[11] Poochanasri M., Lertsakulbunlue S., Kookanok C., Rangsin R., Kaewput W., Mungthin M., Samakkarnthai P. (2025). Triglyceride to high-density lipoprotein ratio as a predictor for 10-year cardiovascular disease in individuals with diabetes in Thailand. J. Health Popul. Nutr..

[12] Liu H., Liu J., Liu J., Xin S., Lyu Z., Fu X. (2022). Triglyceride to High-Density Lipoprotein Cholesterol (TG/HDL-C) Ratio, a Simple but Effective Indicator in Predicting Type 2 Diabetes Mellitus in Older Adults. Front. Endocrinol..

[13] Wang L., Yan N., Zhang M., Pan R., Dang Y., Niu Y. (2022). The association between blood glucose levels and lipids or lipid ratios in type 2 diabetes patients: A cross-sectional study. Front. Endocrinol..

[14] Huang Z., Zhang X., Sun D., Yu K. (2024). Triglyceride to high-density lipoprotein cholesterol ratio is associated with diabetes incidence in non-obese individuals with normoglycemia: A retrospective cohort study based on individuals from East Asia. Front. Endocrinol..

[15] Chauhan A., Singhal A., Goyal P. (2021). TG/HDL Ratio: A marker for insulin resistance and atherosclerosis in prediabetics or not?. J. Fam. Med. Prim. Care.

[16] Jin J., Wang F., Chen S., Shao J., Chen B., Lin X. (2026). Atherogenic Index of Plasma Predicts the Onset and Progression of Cardio-Renal-Metabolic Multimorbidity: Evidence from a Nationwide Prospective Cohort Study. J. Diabetes.

[17] Ostfeld R., Mookherjee D., Spinelli M., Holtzman D., Shoyeb A., Schaefer M., Doddamani S., Spevack D., Du Y. (2006). A triglyceride/high-density lipoprotein ratio ≥3.5 is associated with an increased burden of coronary artery disease on cardiac catheterization. J. Cardiometab. Syndr..

[18] Yeh W.C., Tsao Y.C., Li W.C., Tzeng I.S., Chen L.S., Chen J.Y. (2019). Elevated triglyceride-to-HDL cholesterol ratio is an indicator for insulin resistance in middle-aged and elderly Taiwanese population: A cross-sectional study. Lipids Health Dis..

[19] Pourhoseingholi M.A., Vahedi M., Rahimzadeh M. (2013). Sample size calculation in medical studies. Gastroenterol. Hepatol. Bed Bench.

[20] Aekplakorn W., Taneepanichskul S., Kessomboon P., Chongsuvivatwong V., Putwatana P., Sritara P., Sangwatanaroj S., Chariyalertsak S. (2014). Prevalence of dyslipidemia and management in the Thai population, National Health Examination Survey IV, 2009. J. Lipids.

[21] Aydin F., Murat B., Murat S., Dağhan H. (2025). TG/HDL-C Ratio as a Superior Diagnostic Biomarker for Coronary Plaque Burden in First-Time Acute Coronary Syndrome. Diagnostics.

[22] Jabeen W.M., Jahangir B., Khilji S., Aslam A. (2023). Association of triglyceride glucose index and triglyceride HDL ratio with glucose levels, microvascular and macrovascular complications in Diabetes Mellitus Type-2. Pak. J. Med. Sci..

[23] Zhang Y., Peng N., Wu D., Zhang M., Zhang Q., Shi L. (2024). A prospective cohort study investigating the association between type 2 diabetes and subsequent cardiovascular events in patients with different blood pressure, HbA1c, and lipid levels. Endokrynol. Pol..

[24] Taskinen M.R. (2003). Diabetic dyslipidaemia: From basic research to clinical practice. Diabetologia.

[25] Li G., Deng M., He G., Jiang Y., Wang Z., Zhou C., Xu W., Feng T., Zeng W., Peng J. (2025). Association of non-high-density lipoprotein cholesterol to high-density lipoprotein cholesterol ratio and atherogenic index of plasma with obstructive sleep apnea. Front. Psychiatry.

[26] Zhao M., Xiao M., Zhang H., Tan Q., Ji J., Cheng Y., Lu F. (2025). Relationship between plasma atherogenic index and incidence of cardiovascular diseases in Chinese middle-aged and elderly people. Sci. Rep..

[27] Kitjanukit S. (2026). Association Between the Triglyceride–Glucose Index and Major Cardiovascular Events in Patients with Prior Ischemic Stroke: A Retrospective Cohort Study. Lampang Med. J..

[28] Babic N., Valjevac A., Zaciragic A., Avdagic N., Zukic S., Hasic S. (2019). The Triglyceride/HDL Ratio and Triglyceride Glucose Index as Predictors of Glycemic Control in Patients with Diabetes Mellitus Type 2. Med. Arch..

[29] Yan Z., Liu Y., Huang H. (2012). Association of glycosylated hemoglobin level with lipid ratio and individual lipids in type 2 diabetic patients. Asian Pac. J. Trop. Med..

[30] Baneu P., Văcărescu C., Drăgan S.R., Cirin L., Lazăr-Höcher A.I., Cozgarea A., Faur-Grigori A.A., Crișan S., Gaiță D., Luca C.T. (2024). The Triglyceride/HDL Ratio as a Surrogate Biomarker for Insulin Resistance. Biomedicines.

